# Prophylactic Procurement of University Students in Southern Ethiopia: Stigma and the Value of Condom Machines on Campus

**DOI:** 10.1371/journal.pone.0060725

**Published:** 2013-04-02

**Authors:** Christopher J. Wells, Abraham Alano

**Affiliations:** 1 Tulane University School of Public Health and Tropical Medicine, Department of International Health and Development, New Orleans, Louisiana, United States of America; 2 College of Medicine and Health Sciences, Hawassa University, Hawassa, Ethiopia; UCL Institute of Child Health, University College London, United Kingdom

## Abstract

**Introduction:**

Risky sexual behavior among Ethiopian university students, especially females, is a major contributor to young adult morbidity and mortality. Ambaw et al. found that female university students in Ethiopia may fear the humiliation associated with procuring condoms. A study in Thailand suggests condom machines may provide comfortable condom procurement, but the relevance to a high-risk African context is unknown. The objective of this study was to examine if the installation of condom machines in Ethiopia predicts changes in student condom uptake and use, as well as changes in procurement related stigma.

**Methods:**

Students at a large urban university in Southern Ethiopia completed self reported surveys in 2010 (N  = 2,155 surveys) and again in 2011 (N =  2,000), six months after the installation of condom machines. Mann-Whitney and Chi-square tests were conducted to evaluate significant changes in student sexual behavior, as well as condom procurement and associated stigma over the subsequent one year period.

**Results:**

After installing condom machines, the average number of trips made to procure condoms on-campus significantly increased 101% for sexually active females and significantly decreased 36% for sexually active males. Additionally, reports of condom use during last sexual intercourse showed a non-significant 4.3% increase for females and a significant 9.0% increase for males. During this time, comfort procuring condoms and ability to convince sexual partners to use condoms were significantly higher for sexually active male students. There was no evidence that the condom machines led to an increase in promiscuity.

**Conclusions:**

The results suggest that condom machines may be associated with more condom procurement among vulnerable female students in Ethiopia and could be an important component of a comprehensive university health policy.

## Introduction

HIV/AIDS remains a major threat to the health and well being of those living in low to middle income countries, in particular those in sub-Saharan Africa, a region with more than two-thirds(69 percent) of the world's HIV/AIDS caseload[1]. One of the sub-Saharan countries hit hardest by the HIV/AIDS epidemic is Ethiopia, where in 2010 there were 1.2 million people, or 1.5 percent of the population, living with HIV/AIDS[2], [3].

In 2010 and 2011, the United States' President's Emergency Plan for AIDS Relief(PEPFAR) provided over $290 million(USD) per year to the Government of Ethiopia to implement a comprehensive HIV/AIDS prevention program that included voluntary counseling and testing programs, prevention of mother to child transmission, an "ABC strategy"(abstinence, be faithful, condom use), and distributing antiretroviral therapy medication for those infected with the virus[4], [5]. From 2012–2017, the Government of Ethiopia will continue to work with the United States through the Agency for International Development (USAID) to implement the $90 million(USD) MULU Prevention Program, the largest program of its kind in Ethiopia focusing on protecting the most at-risk populations from HIV/AIDS[6].

The World Health Organization's HIV/AIDS prevention efforts highlight the need to reduce new infections among young adults[7]. Similarly, the Ethiopian Ministry of Health indicates that young urban students, ages 15–24, are the demographic most at risk for acquiring HIV/AIDS, and there is a major gap in student HIV/AIDS prevention services [8]. According to the Kaba study [9] and unpublished observations from our *HIV/AIDS Perceptions of University Students in Ethiopia Survey* (HAPUSES) used as baseline measures for the present longitudinal study (Wells and Alano 2010), about half of sexually active students attending large urban, Ethiopian universities report having concurrent sexual relationships, and about two in five of these students report no condom use during their last sexual experience. Although some students are using condoms, research at a Kenyan University shows there is remarkable discrepancy between students knowing the importance of condom use and the practice of actually using condoms[10].

Unprotected sex among Ethiopian college students is leading to unwanted pregnancy and high drop-out rates among the female population. Registrar reports from Hawassa University (HU), the largest urban school in southern Ethiopia, reported a drop-out rate nearly two times higher for female students in 2010. The HAPUSES indicated that pregnancy was one of the leading reasons females drop-out(Wells and Alano 2010) and the literature indicates that female drop-outs may be led to prostitution, and such reported cases are increasing[11]. Female students are two to four times more likely to contract HIV/AIDS than men, and collegiate responses to HIV/AIDS need to incorporate policies and sanctions that safeguard women [12]–[14]. The benefits of protecting women through family planning, including condom use, range from increased family savings and productivity, to better prospects for employment and ultimately, an improvement in the status of women[15].

Policies encouraging correct and consistent use of condoms can reduce HIV transmission[16], [17]. Results from the 2011 Ethiopian Demographic and Health Survey(DHS) reveal that 61.6% of females and 47.2% of males ages 15–24 with more than one sexual partner in the previous twelve months reported using a condom during their last sexual intercourse. The percentages for this statistic decreased with age, as 47.0% of women and 15.5% of men between ages 15–49 reported condom use during their last intercourse[18].

In an overall condom literature review from 1993–2007, several social factors are associated with unprotected sexual intercourse, including gender inequality, lack of a dialogue among partners about condom use, and the stigma attached to condoms[19]. Despite efforts to increase condom use in Ethiopia, the stigma associated with condom procurement remains a significant barrier to condom uptake. Research at Jimma University in Ethiopia found that stigma associated with condoms was prevalent among students, especially female students[20]. In *Understanding and Challenging HIV Stigma*, the authors assert that condoms provide evidence of an individual's sexual behavior, and sex out of wedlock is often linked with sin[21]. To mitigate this stigma, interventions should reach people where they live, especially women and girls, getting condoms into the hands of those that want them[22].

Ethiopian prevention activities often struggle to safeguard college students from HIV/AIDS due to the heavy stigma involved with procuring condoms. In 2010, the HU health center periodically provided free condoms in small buckets outside the office during the day; however, the HAPUSES reveals that stigma was keeping students, especially females, away from the university health center to otherwise obtain prophylactics(Wells and Alano 2010). One such female student writes, “I don’t pick condoms up from the health center because I don’t want to be humiliated.” Many other students report having to walk a considerable distance off campus to procure condoms from street vendors, community stores, and pharmacies. Other research shows female students, unlike males, reluctantly ask their friends for condoms for fear of being gossiped about [23]. Because students are often incredibly image conscious and concerned with the stigma associated with condom procurement, many prefer anonymous points of condom uptake where a visit to a health facility can be avoided. [24]–[27].

The literature into condom use suggests that selling condoms out of unattended machines may lead to more condom uptake by students. The Yuntadilok study in Thailand suggests condom machines may provide comfortable and easy condom procurement in a community [24]. The price of condoms, however, should also be considered. The Meekers study in Botswana reveals that selling a condom, even at a small price, may create the perception that the condom is of higher quality[23]. Location must also be considered, as a study conducted in Zambia showed that a condom distribution and peer education intervention targeting places where people meet new sexual partners, such as the university, resulted in a substantial increase in reported condom use [28]. These lessons of improving perceptions of condom quality and accessibility can be used to fill a vital research gap in Ethiopia, where public health officials strive to learn innovative ways to reduce the stigma associated with condom procurement[29]. To that end, new interventions tailored to Ethiopian university students must improve the students' attitudes towards condoms to create protective behavior change[29].

The current longitudinal study will supplement the narrow field of condom machine research to investigate condom procurement and the related stigma in a high-risk Ethiopian population. Our previous baseline investigation using HAPUSES will be repeated to measure accessibility of prophylactics on campus, if risky sexual behavior (unprotected vaginal sex) changed over the one year period (2010–2011), and whether introduction of discreet condom dispensers related to changes in condom procurement and stigma. We hypothesized that female students, who are more concerned with stigma associated with procuring a condom, would report higher levels of condom uptake and use over time.

## Methods

This longitudinal study was comprised of two series of the same HAPUSES, conducted one year apart (April 2010 and April 2011), which assessed the sexual behavior and HIV/AIDS perceptions of the 9,568 students living in the residential halls of Hawassa University. HU had a total of 13,653 students in 2010 and 15,211 students in 2011. The study received ethical approval from the Ethiopian government's Southern Nations, Nationalities, and People’s Regional Health Bureau, the Hawassa University Ethical Committee, and the Tulane University Institutional Review Board.

Students living in the university dormitories were recruited because they rely most on the reproductive health services of the university. The surveys were entirely self-administered, handed out and collected by the researchers and eleven volunteer health care professionals (each given ethical training and specific job expectations by the researchers) in person on an individual basis, to a random sample of students. The surveys were written in English because the entrance exams and classes at the university are given in English. Each survey was completely anonymous and contained no information that could be used to directly identify a particular student. Each year, recruitment was organized using a computer driven random number generator to select the order of each of the 25 different residential halls(six female halls and nineteen male halls) on campus to be surveyed, where researchers attempted to sample every third student walking into or out of the building; a strategy employed to include only students living on-campus. The students were surveyed at three different times: 9 AM, 1 PM, and 6 PM at 30 minute intervals until 85 surveys were collected from each residential hall. All students included in the study provided informed consent.

The first HAPUSES survey was administered Monday through Friday for the first two weeks of April 2010 (T1), and similarly re-administered in April 2011 (T2) after all three condom machines(total) had been installed for six months. The locations of the machines were strategically chosen to provide a combination of privacy, protection from the sun, and maximum foot traffic: outside the main centrally-located restroom shared by the male residents, outside the main centrally-located restroom shared by the female residents, and outside the health center. A small sticker was applied to each of the condom machines with a description(in English) of the efficacy of condoms in preventing pregnancy and sexually transmitted infections(STIs), including HIV/AIDS. Each machine dispensed only "Sensation" Brand male condoms, the same condoms freely available at the HU health center. The timing of the survey was chosen so as not to conflict with the students' vacation and final exam period. The participants were given no incentives to take part in the research and were reassured that their answers would remain anonymous. Additionally, participants were asked if they had previously taken the survey in the past week, and those that indicated they had were ineligible to participate. The last question on the survey asked the participant if they had answered all of the questions honestly. Only honest responses were used in the study. Participants completed the surveys in each dormitory entrance area, where surveys were subsequently collected in a box held by the researcher. In the 2010 survey, there was a 68% response rate for the 2,155 surveys collected, with 71 students refusing to participate. This sample size was comparable to the 2,000 surveys collected in 2011, of which there was a 65% response rate and 64 students refusing to participate. The 2010 and 2011 surveys had 98.1% and 96.2% of the students report honest responses, respectively.

### Measures

Students responded to 31 survey items. The first six items evaluated the demographics of the sample. Six items assessed their perceptions of condom procurement (items were scaled 1 to 5: where 1 =  “very difficult”, 2 =  "difficult", 3 =  "neither difficult or easy", 4 =  "easy", and 5  =  “very easy”) and stigma associated with condom use and procurement(items were scaled 1 to 5: where 1 =  “very uncomfortable”, 2 =  "uncomfortable", 3 =  "somewhat comfortable", 4 =  "comfortable", and 5  =  “very comfortable”). Six items assessed the students' measure of confidence in condoms (items were scaled 1 to 5: where 1 =  “not confident” 2 =  "slightly confident", 3 =  "moderately confident", 4 =  "confident", and 5  =  “very confident”) in protecting against pregnancy and STIs, including HIV/AIDS. We also assessed risky sexual behavior with seven items about frequency of condom use (items were scaled from 1 to 5: where 1 =  “never”, 2 =  "sometimes", 3 =  "regularly", 4 =  "frequently", and 5  =  “always”), whether a condom was used during the last sexual intercourse, and items evaluating sexual debut, as well as the number of sexual partners in the past six months. In addition, we asked students about concurrent sexual relationships, how many trips were made for condoms off campus (street vendors, communities stores, and pharmacies), trips for condoms on campus, and how often they perceived themselves to be potentially exposed to HIV/AIDS (1 =  “never” to 5  =  “always”) by engaging in risky unprotected sex. Questions concerning the number of sexual partners and concurrency were added to address fears among some Hawassa University administrators that condom machines may increase promiscuity. Also, there is a low expression of homosexuality in Ethiopia, along with laws that forbid it, and therefore questions investigating homosexual activity were avoided.

Because the effectiveness of condom machines is a relatively under-researched area, no existing instruments exist whose psychometric properties have been tested. Thus, the scales in this survey are new. Before entering the field with the questionnaire, individual questions were discussed with local university students to ensure cultural sensitivity and to verify that the questions were valid measures of what we were seeking to understand. For this analysis, only a subset of the survey questions is considered. Specifically, we compare average scores before and after condom machine installation on whether a respondent used a condom at the last sexual encounter (yes/no); if the respondent owned a condom, either on their person or in their dormitory(yes/no); the number of sexual partners; comfort purchasing condoms and visiting the health care facility (1 =  “very uncomfortable” to 5  =  “very comfortable”); and frequency of on-campus condom trips. In addition, questions related to the perceived effectiveness of condoms against pregnancy, STIs, and HIV are considered, along with confidence in using a condom. Although these four items belong to the larger scale “confidence in condoms” scale (Cronbach’s alpha  =  .817), they are analyzed separately here to better understand the difference in specific beliefs.

### Data analysis

The questionnaire data for both years was manually entered into Microsoft Excel. The two spreadsheets were compiled, and Statistical Package for Social Sciences (SPSS Inc., Chicago, IL, USA, version 15.0) software was used for analysis. Two data samples, T1 and T2, looked at gender, age group (18–19 yrs, 20–21 yrs, 22–23 yrs, 24+ yrs), birth place (rural, small town, city), and year of school (first year/freshman, second year/sophomore, third year/junior, fourth year or higher/senior or graduate student) for each residential hall. Mann-Whitney and chi-square tests were then conducted to determine if comfort in condom procurement, confidence in effectiveness of condoms, accessibility of condom supply, number of sexual partners in past six months, number of concurrent partners in past six months, perceived potential exposure to HIV/AIDS in past six months, frequency of condom use in past six months, and uptake of campus condom supply in past six months had significantly changed after the installation of the condoms machines. The Mann-Whitney test is a non-parametric version of the t-test that was employed in cases when the dependent variable was measured on an ordered scale rather than an interval scale. When the dependent variable was dichotomous or nominal, chi-square tests were used. The interval-level measures, such as counts of sexual partners, deviate strongly from normality and hence were also analyzed using non-parametric methods. Behavior trends were also analyzed through a stratified chi-squared analysis based on gender, age, birthplace, and year of study.

An a priori power analysis was carried out using nQuery software (nQuery Advisor 7.0, Statistical Solutions, USA). For the Mann Whitney tests, the effect size assumed that 5% of the condom abstainers would become condom users after the implementation of the condom machine. Under this scenario, a sample size of 780 would be necessary in each group (pre and post) to obtain a power of .80. Observing a small effect size (*w*  =  .1) for a chi-square test with one degree of freedom requires a sample size of 785 to obtain a power level of .8; a chi-square test with three degrees of freedom requires a sample size of 1,090. The sample size is therefore large enough to pick up relatively small changes in behaviors and attitudes following the installation of the condom machines.

Each of the two years represented a separate cohort, so the two samples (T1,T2) are treated as independent. Although a form of random cluster sampling was used (choosing dorm first, then sampling within the dorm), mixed model analyses showed that the percentage of variance attributable to the dorm level was 5% or less. Hence, the analysis below treats all observations as independent.

## Results

### Sample Demographics

In 2010 and 2011, 22.5% and 20.9% of the students living in the dormitories were randomly sampled, respectively. This sampling equated to 15.8% of the total student population for 2010 and 13.1% for 2011. Most of the sampling was freshman or sophomore males (see Table 1) of 20–21 years of age, most commonly born in a rural area, and all of the students reported living in the residential halls on campus. The 2010 student sample (N = 2,155) had fewer freshmen and more senior students, and a larger proportion reported honest answers on the surveys.

**Table 1:** Demographic characteristics of Hawassa University and student samples, 2010 and 2011.

	*2010 population*		*2011 population*		*2010 sample*		*2011 sample*	
	*(N = 13,653)*		*(N = 15,211)*		*(N = 2,155)*		*(N = 2,000)*	
*Characteristic*	*N*	*%*	*N*	*%*	*n*	*%*	*n*	*%*
Gender								
Male	11,137	81.6	12,669	83.3	1,647	76.4	1,552	77.6
Female	2,516	18.4	2,542	16.7	508	23.6	448	22.4
Birthplace								
Rural Village	–	–	–	–	609	28.4	626	31.3
Small Town	–	–	–	–	546	25.5	595	29.8
Large Town	–	–	–	–	426	19.9	319	19.5
City	–	–	–	–	561	26.2	450	22.5
Age								
18–19 years	–	–	–	–	412	19.2	319	19.6
20–21 years	–	–	–	–	1,237	57.7	859	52.7
22–23 years	–	–	–	–	430	20.1	363	22.2
24+ years	–	–	–	–	65	3.0	88	5.4
Year in School								
Freshman	3,742	27.4	5,411	35.6	801	37.3	896	44.9
Sophomore	6,162	45.1	3,421	22.5	886	41.3	383	19.2
Junior	2,951	21.6	5,445	35.8	196	9.1	521	26.1
Senior	798	5.8	934	6.1	263	12.3	197	9.9
Honest responses on survey								
No	–	–	–	–	41	1.9	76	3.8
Yes	–	–	–	–	2109	98.1	1924	96.2

Chi-square tests were run to determine if any demographic differences between the two cohorts were statistically significant. Given the sample consists of over 4,000 subjects, the power of the test is extremely high and hence is likely to find even small changes to be significant. The difference in the distribution of gender was not statistically significant (χ^2^  =  .806, df  =  1, *p*  =  .369), but the tests for age, year, and birthplace all yielded p-values less than .001. Looking more closely at the percentages in Table 1, it is clear that the only sharp change was in the number of sophomores and, relatedly, the number of 20 to 21-year olds. Both declined by more than 5% in 2011 relative to the categories, both younger and older. Previous research has shown that university students tend to evolve over the course of a four-year period of study, which suggests that the underrepresentation of sophomores may impact the results[30], [31]. Nonetheless, assuming that growth trajectories are not radically nonlinear, the large number of freshmen and junior subjects should average over the underrepresented sophomores to provide valid estimates of the overall effectiveness of condom machines among all students.

### Trends in Student Sexual Behaviors

The total sample size was 2,155 at T1 and 2,000 at T2, but this includes students who reported that they had not been entirely honest on the survey as well as those who were not sexually active. The analysis that follows excludes surveys when the respondent checked that he or she had not been totally honest or had not had any sexual partners. This resulted in a sample size of 457 to the 2010 survey and 459 for the 2011 survey. Table 2 displays the changes in percentages in sexual behaviors for the categorical variables, and Table 3 shows changes in average scores on the ordered and interval-level variables. Both tables report percentages for the subsample of honest and sexually active respondents. Listwise deletion was used for missing data within the subsample. Table 2 reports N for sexually active, honest responses with complete observations on the respective variables.

**Table 2:** Comparisons of 2010 and 2011 Hawassa University student sexual behavior.

		*2010 sample*	*2011 sample*	*Chi-squared*
		*(N = 457)*	*(N = 459)*	*statistic (df)*
Condom use during last sexual intercourse		61.8(%)	71.4(%)	9.53(1)
		(N = 281)	(N = 327)	
Males		65.2	74.2	7.59(1)
		(N = 247)	(N = 302)	
Females		44.7	49	0.23(1)
		(N = 34)	(N = 25)	
Student reported owning a condom(on person or in dormitory) at time of survey		45.4	41.9	1.08(1)
		(N = 182)	(N = 190)	
Males		47.9	43.2	1.65(1)
		(N = 160)	(N = 174)	
Females		32.8	31.4	0.028(1)
		(N = 22)	(N = 16)	
Any concurrent sexual partners in past six months		56.7	42.5	7.60(1)
		(N = 246)	(N = 192)	
Males		61.5	44.0	8.34(1)
		(N = 219)	(N = 177)	
Females		34.6	30.0	0.29(1)
		(N = 27)	(N = 15)	

Note: Sample is limited to respondents who reported having had at least one sexual partner in past six months and reported having answered the survey honestly.

**Table 3:** Hawassa University student descriptive statistics for ordered and interval-level dependent variables, 2010 and 2011.

	Minimum	Maximum	Mean	Std. Deviation
	Year = 2010			
Confident Use Condom Correctly?	1	5	3.43	1.226
Condom Effective against Pregnancy?	1	5	3.64	1.073
Condom Effective against STIs?	1	5	3.20	1.158
Condom Effective against HIV?	1	5	3.30	1.128
How Many Sexual Partners?	1	20	2.14	1.928
Use Condom how Often?	1	5	2.96	1.477
Perceived Exposure to HIV?	1	5	1.71	0.994
Comfort Procuring Condoms on Campus?	1	5	2.73	1.462
Difficulty Obtaining Condoms on Campus?	1	5	3.13	1.484
# Trips on campus for Condoms?	0	20	1.55	2.631
# Trips off Campus for Condoms?	0	30	1.83	3.367
Comfort Convincing Partner to use Condom?	1	5	3.32	1.397

	Year = 2011			
Confident Use Condom Correctly	1	5	3.92	1.356
Condom Effective against Pregnancy?	1	5	3.94	1.216
Condom Effective against STIs?	1	5	3.73	1.276
Condom Effective against HIV?	1	5	3.45	1.318
How Many Sexual Partners?	1	20	2.05	1.855
Use Condom how Often?	1	5	3.09	1.674
Perceived Exposure to HIV?	1	5	1.97	1.406
Comfort Procuring Condoms on Campus?	1	5	3.44	1.595
Difficulty Obtaining Condoms on Campus?	1	5	3.04	1.614
# Trips on Campus for Condoms?	1	2	1.66	0.475
# Trips off Campus for Condoms?	0	24	2.3	3.584
Comfort Convincing Partner to use Condom?	1	5	3.44	1.595

Note: Sample is limited to respondents who reported having had at least one sexual partner in past six months and reported having answered the survey honestly.

Between 2010 and 2011, there was a significant overall increase in condom use reported for students during their last sexual experience (61.8%, or 281 of 455 sexually active and honestly responding subjects, vs 71.4%, or 327 of 458 sexually active and honestly responding subjects, Chi-Square  =  9.53, df = 1, *p*  =  .002), most specifically for male students (65.2%, or 247 of 379 sexually active and honestly responding males, vs 74.2%, or 302 of 407 sexually active and honestly responding males, Chi-Square  =  7.59, df = 1, *p*  =  .006) that showed a significant 9.0% increase, but not for female students (44.7%, or 34 of 76 sexually active and honestly responding females, vs 49%, or 25 of 51 sexually active and honestly responding females, Chi-square  =  0.23, df = 1, *p* =  .635) that showed a non-significant 4.3% increase in condom use at last sexual intercourse. Frequency of condom use increased on average among all sexually active respondents, with an average response of 2.96 in 2010 moving to 3.09 in 2011, though this was not statistically significant (Z  =  –1.195, *p*  =  .232). Looking specifically within genders, there was a moderate, though non-significant, increase in condom usage among sexually active males, with average responses changing from 3.02 to 3.21 (Z  =  –1.74, *p*  =  .082). For sexually active females, there was a statistically significant decline in frequency of condom usage, with average responses changing from 2.68 to 2.20 (Z  =  –2.11, *p*  =  .035). There was no significant change in the amount of respondents reporting condom ownership (45.4%, or 182 of 401 sexually active and honestly reporting respondents, vs. 41.9%, or 190 of 454 sexually active and honestly reporting respondents, Chi-Square  =  1.08, df = 1, *p*  =  .298).

The data indicates there was no significant change in students' perceptions of possible exposure to HIV/AIDS for those sexually active in the past-six-months ( _2010_ =  1.71, µ_2011_ =  1.97, Z  =  –76, *p*  =  .447) or for any demographic subgroup examined (gender, age group: 18–19 yrs, 20–21 yrs, 22–23 yrs, 24+ yrs, birth place: rural, small town, city, year of school: freshman, sophomore, junior, senior/graduate student).

There also was a non-significant decrease in the overall number of sexual partners reported from 2010 to 2011 (Mean  =  2.14 vs 2.05, Z  = –1.628, *p*  =  .104). In subgroup analyses, both the male(Mean  =  2.22 vs 2.1, Z  =  –1.884, *p*  =  .060) and female (Mean  =  1.74 vs 1.65, Z  =  –511, *p*  =  .610) students had a non-significant decrease in the number of sexual partners. Similarly, the number of sexually active and honest respondents reporting concurrent partners in the past six months significantly decreased overall (56.7%, or 246 of 434 sexually active and honest respondents, vs 42.5%, or 192 of 452 sexually active and honest respondents, Chi-Square  =  7.60, df = 1, *p*  =  .006) and specifically for males (61.5%, or 219 of 356 sexually active and honestly reporting males, vs. 44%, or 177 of 402 sexually active and honestly reporting males, Chi-Square  =  8.34, df = 1, *p*  =  .004) reporting sexual activity in the past six months, but not among female students (34.6%, or 27 of 78 sexually active and honestly reporting females, vs. 30.0%, or 15 of 50 sexually active and honestly reporting females, Chi-Square  =  .29, df = 1, *p*  =  .587).

### Trends in Stigma and Condom Procurement

Students who were sexually active in the last six months indicated they were significantly more comfortable procuring condoms on campus (µ_2010_ =  2.73, µ_2011_ =  3.44, Z  =  –6.55, *p* < .001) after the installation of condom machines. The subgroup analyses revealed significant improvements in the male students' comfort in procuring condoms (µ_2010_ =  2.75, µ_2011_ =  3.5, Z  =  –6.54, *p* <.001), but only a minor non-significant increase among female students (µ_2010_ =  2.65, µ_2011_ =  2.98, Z  =  –0.90, *p*  =  .370). Those reporting sexual activity in the past six months also reported a slight overall decrease in the difficulty obtaining condoms on campus (µ_2010_ =  3.13, µ_2011_ =  3.04, Z  =  –75, *p*  =  .455) between years, though not significant.

The school nurses reported selling an average of 42 condoms from each condom machine per week. Self reported uptake of the university condom supply increased significantly over time for female students reporting sexual activity in the past six months (µ_2010_ =  .87, µ_2011_ =  1.75, Z  =  –3.199, *p* < .001), a 101% increase, but decreased significantly for males (µ_2010_ =  1.71, µ_2011_ =  1.65, Z  =  –5.202, *p* < .001), a 36% decrease. There was no significant change in the uptake of condoms off-campus for this group of females.

There was a significant increase (µ_2010_ =  3.32, µ_2011_ =  3.44, Z  =  –2.06, *p*  =  .039) in sexually active students reporting they could convince their sexual partner to use a condom. There was a gender interaction for this question, with a significant increase over time for male students (µ_2010_ =  3.29, µ_2011_ =  3.5, Z  =  –2.778, *p*  = .005), but not for female students (µ_2010_ =  3.47, µ_2011_ =  2.98, Z  =  –1.329, *p*  =  .184).

### Trends in Condom Perceptions

Trends were also evaluated for sexually active and honest respondents to identify changes among this subset of the sample before and after the installation of the condom machines. The students' overall evaluations considering the effectiveness of condoms against pregnancy (µ_2010_ =  3.64, µ_2011_ =  3.94, Z  =  –5.247, *p* < .001), STIs (µ_2010_ =  3.20, µ_2011_ =  3.73, Z  =  –7.203, *p* < .001), and HIV/AIDS(µ_2010_ =  3.3, µ_2011_ =  3.45, Z  =  –2.589, *p*  = .010) all increased significantly over the two survey years. These changes, however, were primarily driven by males. Females did not exhibit a significant change in beliefs regarding pregnancy (µ_2010_ =  3.46, µ_2011_ =  3.73, Z  =  –1.589, *p*  = .112) or HIV ( µ_2010_ =  3.18, µ_2011_ =  3.45, Z  =  –1.517, *p*  = .129). Male students that were sexually active in the past-six-months also reported more confidence in their ability to use a condom correctly over time (µ_2010_ =  3.42, µ_2011_ =  3.99, Z  =  –7.916, *p* <.001).

## Discussion

The data shows self-reported condom use during the students' last sexual intercourse increased significantly for males and non-significantly for females following the installation of condom machines at Hawassa University. Compared to the 2011 HU data, the Ethiopian DHS(2011) reports 13% more condom use for females and 27% less condom use for males(ages 15–24) on this measure[18]. The lower numbers of condom use reported for the female HU students reinforces the need for more innovative interventions to reach this demographic and may hint at the prevalence of a gender related stigma towards condoms on campus.

For the female students, whom the university has targeted as the most vulnerable demographic for acquiring HIV/AIDS, the results indicated a large significant increase in the uptake of the on-campus condom supply. Additionally, there was a non-significant increase in comfort in obtaining condoms on campus. We hypothesize that condom machines may have reduced the stigma associated with condom procurement by allowing these female students a chance for anonymous uptake at any time.

The data indicates there are factors inhibiting condom use, or reporting condom use, amongst females. Despite a slight increase in the percentage of females reporting condom use during their last sexual intercourse and increased condom uptake, we found a decrease in the overall frequency of condom use reported amongst sexually active females. Because data indicates females believe condoms are effective in preventing STIs, the reduction in condom use amongst females could be attributed to longer monogamous relationships in which females may not be worried as much about STIs. Indeed, the results for 2010 show fewer females reporting only one partner when compared to 2011 (59% vs 65%). The notion that monogamy among female students may be effecting condom use is supported by research at Debre Birhan University in Ethiopia where females students in a trusting relationship were less likely to use a condom [32]. Additionally, research out of West Africa supports the notion that condom use may be underreported as condom procurement is highly stigmatized, creating a social desirability bias that may have encouraged female students to respond defensively to this survey question with hopes of protecting their reputation, and that of their university[33].

An unintended benefit of the research was that it started a conversation about reproductive health amongst students and administrators at the university, thus raising awareness on-campus. Such a phenomenon is unique in an indirect Ethiopian culture that often avoids such personal topics and may be an important step towards reducing stigma related to condom procurement amongst students. After the condom machines were installed, male students perceived condoms to be more effective and were more comfortable convincing their partners to practice safe sex. This is reasonable given the responses from the initial HAPUSES reporting the previous manner of dispersing condoms on campus was via condom buckets that were infrequently placed outside the health clinic, and that students reported that many of the condoms were expired and left in the sun, leaving them to question their efficacy(Wells and Alano 2010).

The intervention was limited to a total of three condom machines because some HU administrators worried that installing condom machines may encourage student sexual activity. The results appear to suggest otherwise, as we found a decrease in the overall number of sexual and concurrent partners reported after the machines were introduced; however, this reduction, especially the 17% reduction in concurrency, cannot be attributed to the machines alone. In 2010, the Ethiopian Ministry of Health implemented its second multi-sectored strategic plan to respond to HIV[3]. This five year plan focuses heavily on abstinence and being faithful, and may also have affected the concurrency rates. Ethiopian public health measures should continue to emphasize the reduction of sexual partners, as an analysis of the AIDS epidemic in Uganda confirmed that reducing the number of sexual partners(including reducing concurrency), in tandem with increased condom use and a delay in age of first sexual intercourse, were all important factors in the decline of HIV prevalence documented there in the 1990's[34], [35].

Safe sex among sexually active students was encouraged by strategically placing condom machines across campus in places that are visible, yet would allow confidential uptake. Each of the machines remained functional during the study and displayed a sticker promoting abstinence or condom use in the prevention of disease and unwanted pregnancy. The stickers may have influenced students by providing a constant reminder of the implications of risky sexual behavior every time the students visited the restrooms. The new condom machines and efficacy stickers signal a more open prophylactic policy by the administration, and though the intervention reached but a fraction of the HU students, our research suggests that clear condom promotion by university authority figures may encourage more condom trust, uptake, and use amongst students, especially among the female demographic[12], [14]. Installing three condom machines may appear small in scale, however, discerning these machines are culturally acceptable in this context opens the possibilities for similar, more substantial interventions.

This study is the first of its kind suggesting that condom machines may provide a new way to reach university students in Ethiopia. However, there is a shortage of supporting research suggesting the benefits of such interventions in other parts of the world. One of the lessons learned from the Yuntadilok study in Thailand was that condom machines were widely accepted to be the most convenient and accessible ways to get condoms discreetly[24]. It is difficult to generalize the results of our findings to other institutions in the country and across the world due to the cultural, civil, and socioeconomic factors that make Ethiopia unique. The high prevalence of HIV/AIDS afflicting Ethiopia may impact the prophylactic procurement behaviors of these students more so than others. Although students reported no change in the difficulty obtaining condoms and did not self report having more condoms in their possession, male students did report significantly more comfort in the condom procurement process. This finding may suggest the same sexually active students were procuring condoms in 2010 and 2011, but experienced less embarrassment doing so in 2011. The difference in our findings may be due to the fact that students will use more condoms if they can readily access them without feeling judged and that they trust the quality of the supply. Meekers et al. in Botswana suggests that condoms sold may be perceived to be of higher quality than free condoms[23]. As such, the simple act of selling condoms via the new machines could be leading students to have more confidence in their condom’s effectiveness. Condom machines are not a panacea for the reproductive health problems found on campus and should be used in conjunction with other education efforts, such as condom peer education clubs, as more comprehensive prevention packages may significantly lower sexual risk and activity, both among those already sexually active and those who are not[36], [37].

This longitudinal study had several strengths; inclusion of a large and random representative sample of vulnerable students that examined myriad behaviors, procurement stigma, and perspectives relating to risky sexual behavior; the use of a limited timeframe that reduced recall bias and possible cultural differences across generations thus making observed trends more accurate; and self administering surveys minimized the impact of a response bias. The study, however, was not without limitations. Despite the random sample surveyed, response bias may have affected the results as the differences between participant and non-participants were not analyzed[38]. Similarly, positivity bias could have been introduced as respondents built rapport with surveyors and tried to please them by providing expected answers. The converse may also be true, as antagonism towards surveyors may have manifested disagreeable responses[39]. We might also consider that the Mann-Whitney tests assume that the two samples are completely independent. That is, the people interviewed at T1 were not the same as those interviewed at T2. Unfortunately, to be able to correct for this, it would be necessary to be able to match the subjects at both time points, which is not possible with the current data that was coded and anonymous to protect the students. However, we assume independence because a large sample of subjects were randomly selected from a population of nearly 10,000 students and were most likely different over time. The random selection methodology for T2 also yielded older students than T1. Older students may have more sexual maturity and may be more likely to procure and use condoms. In addition, our sample was only taken from one university, limiting our ability to examine regional trends and environmental confounders (e.g. local NGO HIV/AIDS counseling and testing programs, community and university education programs, condom marketing campaigns, government regulations and policies) that could influence the students' responses. Also, the nurses tasked with maintaining the condom machines reported regularly refilling the condom supply on a weekly basis, though there is no certainty that condoms reportedly used during this period were purchased from the machines. Subsequently, we caution against making any direct inferences about the cause and effect relationship of the results.

Since this is novel research, and especially given the cultural sensitivity of the topic, researchers wanted to focus on a smaller intervention serving a subpopulation of students before making recommendations to the larger population of students. As such, future research examining prophylactic procurement could include more condom machines, other universities without condom machines, include direct quantitative data of condom uptake from different locations, survey school nurses, analyze medical records (e.g., pregnancy and STI trends), and account for community programs that are promoting reproductive health among students.

## Conclusions

Our study results suggest that the installation of condom machines at large universities in Ethiopia, may perhaps be a new and effective way to increase the uptake of condoms among a vulnerable demographic of female students traditionally intimidated by the stigma associated with condom procurement. A concerted effort on the part of university and government administrators may thwart unwanted pregnancy and HIV/AIDS transmission through policy that unabashedly promotes education, awareness, and prevention programs. The results of our study stress the importance of creating innovative and consistent ways to reduce stigma associated with condom procurement on campus and has indicated that students respond positively to such ideas. Further efforts must continue to address the health and well-being of the university students, particularly, the female students, for they embody the future of Ethiopia.
